# Perspectives on Challenges to Cell Therapy Development in Taiwan: Strengthening Evidential Standards and Ways Forward

**DOI:** 10.3389/fbioe.2021.789043

**Published:** 2021-12-16

**Authors:** Bilikis Aderonke Abolarinwa, Malissa Kay Shaw, Chung-Hsi Lee

**Affiliations:** ^1^ International PhD program for Cell Therapy and Regeneration Medicine, College of Medicine, Taipei Medical University, Taipei, Taiwan; ^2^ Graduate Institute of Humanities in Medicine, Taipei Medical University, Taipei, Taiwan; ^3^ School of Nursing, College of Nursing, Taipei Medical University, Taipei, Taiwan; ^4^ Graduate Institute of Health and Biotechnology Law, Taipei Medical University, Taipei, Taiwan

**Keywords:** cell-based therapy, regenerative medicine, regulation, qualitative research, clinical evidence, Taiwan

## Abstract

Over the past years, the field of regenerative medicine and cell therapy has garnered much interest, extending beyond the bench to broader use, and commercialization. These therapies undergo stringent regulatory oversight as a result of their complexities and potential risk across different jurisdictions. Taiwan’s government, with the aim of developing the country as a hub for regenerative medicine in Asia, enacted a dual track act to promote the development of regenerative and cell therapy products. This qualitative study used purposive sampling to recruit sixteen experts (Twelve respondents from medical institutions and four respondents from the industry) to understand their perspectives on one of the regulatory tracks which governs the medical use of cell technologies and challenges regarding its implementation. Semi-structured interviews were conducted, transcribed, coded and thematically analyzed. Three major themes emerged from the analysis: 1) Perceptions of the “Special Regulation for Cell Therapy” 2) Emerging issues and controversies on the medical use of cell technologies in private clinics, and 3) Challenges impeding the clinical innovation of cell technologies. As reported by the experts, it was clear that the special regulation for cell therapy was aimed at legalizing the clinical use of cell therapy in a similar fashion to an evidence-based pathway, to promote clinical innovation, ensure manufacturing consistency, and improve oversight on cell-based therapies. Thus, the regulation addresses the issues of safety concerns, patient’s access and stem cell tourism. However, the limited approved cell techniques, quality control during cell processing, time, and criteria used in evaluating applications in addition to the need to develop evidential standards for clinical evidence are some of the difficulties faced. Thus, policy interventions on funding, educational resources, training, and regulatory clarity addressing these challenges may positively impact clinical innovation of cell therapy in Taiwan.

## 1 Introduction

Regenerative medicine products are designed to replace or regenerate a specific target site to normal function, and in some conditions provide a cure. Cell-based therapies (CBT) are a subset of regenerative products composed of living cells which are transplanted into a donor or recipient to address unmet medical needs (Petricciani et al., 2017). The potential of these therapies has extended beyond scientific research to commercialization and broader market use. Hence, regulatory bodies across jurisdictions have continually established and published regulations and guidelines to evaluate CBT based on their perceived risk, and have utilized a risk-based classification style to categorize them as either medical procedures or biological drug products (FDA, 2017; A, (2008). EMA, 2008; PMDA, 2004).Taiwan’s cell therapy regulation is harmonized with that of the United States and Europe. However, in order to promote patient’s access to CBT due to demands for innovative treatments as well as the interest of cell therapy developers, the government adopted a dual track regulation for CBT products from Japan in 2018. The framework includes the “Regulation Governing the Application of Specific Medical Examination Technique and Medical Device” (RASMET) also known as the “Special Regulation for Cell Therapy” under the medical care act and “Regenerative Medicinal Product Management Act” solely under the pharmaceutical affairs act (CHEN, 2019; Tsai et al., 2020) which jointly promote the development of regenerative medicine. The “Special Regulation for Cell Therapy” was designed to govern the use of CBT as a technique for medical practice to promote clinical innovation across approved medical institutions for patients with specific medical indications. Thus, medical institutions need to fulfill these requirements:

1) Certification of operating physicians who are specialists in the field of the diseases for which cell therapy is to be applied.
2) The completion of training courses on regulations, ethics, cell processing unit management, and adverse event reporting by the operating physicians.
3) The physician’s previous participation in human clinical trial for the specific cell therapy technique intended for application.

Furthermore, institutions are required to hold the certification of standardized GTP (Good Tissue Practice) facility. Few medical institutions own a standardized facility while others collaborate with and obtain processed CBT products from GTP facilities owned by industries. In addition, a treatment plan is also required, all of which needs to be submitted to the regulatory authority, the Ministry of Health and Welfare (MoHW). Although, industries process these products. Both medical institutions and physicians are responsible for the risk of these products, and are thus considered medical technologies under the special regulation.

**FIGURE 1:**
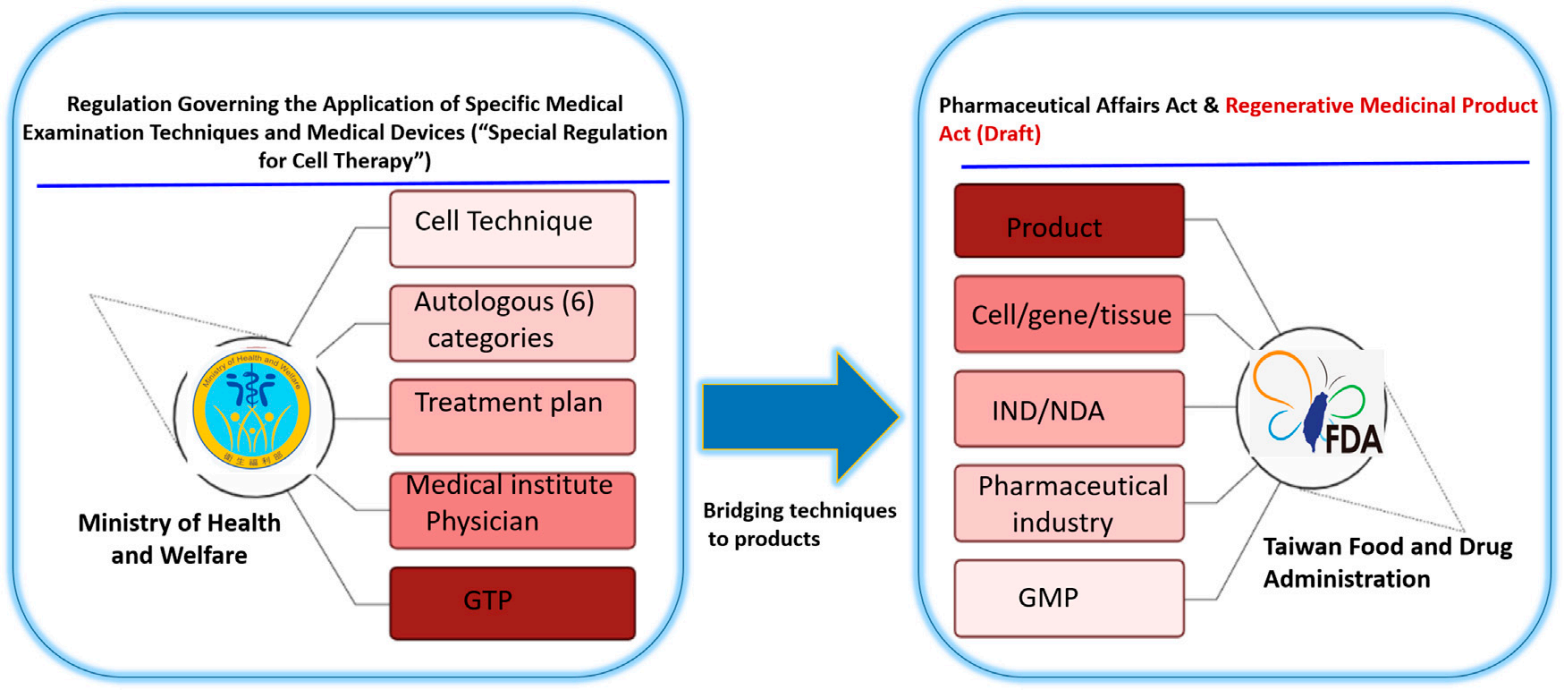
Taiwan’s dual track regulatory framework. The special regulation permits the use of six types of autologous cell techniques in medical institutions by approved physicians under the Good Tissue Practices (GTP), while the regenerative medicinal act permits the manufacturing of medicinal products by pharmaceutical industry under the Good Manufacturing Practices (GMP).

**FIGURE 2:**
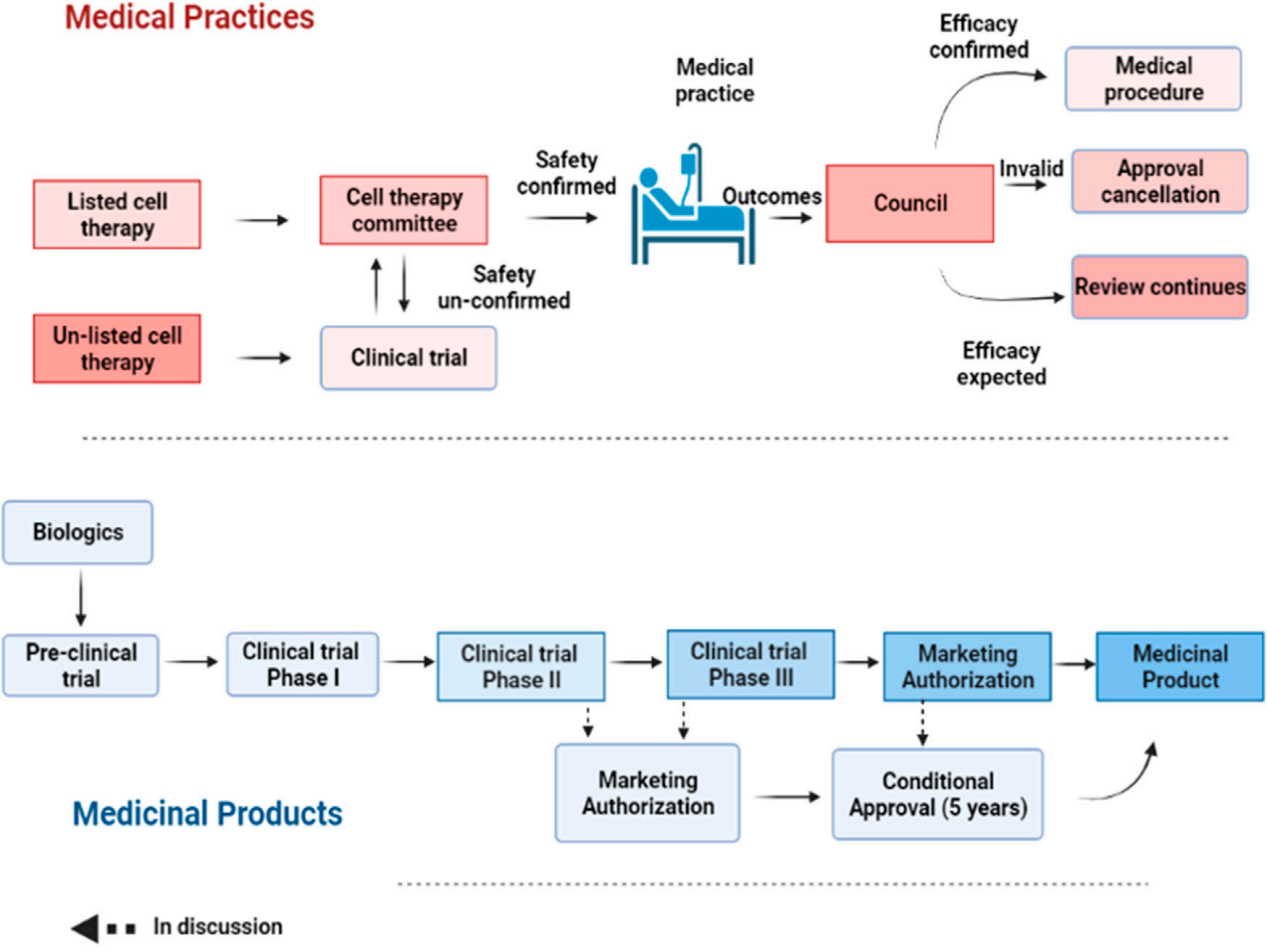
A step-wise process of approval for medical practice and products. For medical practice, the safety of a listed cell technology is reviewed by the cell therapy committee for approval. Once its efficacy is confirmed, such cell technology can be turned into a routine practice. Otherwise, it will be annulled. For unlisted cell technology, it needs to undergo clinical testing before being approved by the committee for cell therapy as a medical practice. Regenerative products or biologics need to undergo a stepwise clinical testing from phase I-III before being granted a market authorization. However, a conditional approval of 5 years is allowed after preliminary safety and efficacy results are provided.

Within the context of this regulation, the MoHW has approved 6 types of autologous CBT (technologies) considered low risk based on their expected/predictable efficacy and accumulated safety profiles for specific indications, and do not require investigational new drug application (IND) market approval (Chen, 2016; CHEN, 2019) (Table 1; Figure 1). However, CBT products (technologies) from autologous sources not included in the approved list requires clinical evidence before being granted an approval (Figure 2). For this purpose, the MoHW established and authorized two review committees to oversee the use of these CBT technologies in medical practice. One of the committees reviews the proposals focusing on the scientific basis and the treatment plan of the specific CBT products while the other committee reviews the consumer price of the CBT treatment. Each of the committees includes experts from scientific, clinical, statistics, legal, and bioethics fields.

**TABLE 1:** Risk-based classification of cell-based therapies.

Medical technologies (low risk)		Medicinal products (high risk)
Cell types	Indication	Product types
Autologous CD34^+^ selection peripheral blood stem cells transplantation	1. Hematological malignancies include leukemia (excluding the chronic phase of chronic myeloid leukemia), lymphoma, and multiple myeloma	Current developing innovative cell therapy products CAR-T products • Welgenaleucel (UWC19) Cell therapy products • PB103 (Allogeneic NK cell) • ADCTA-SSI-G1 (Dendritic cell/tumor antigen) • CLS2702C/CLS2702D (Autologous oral mucosal sheet)• NK+ NKT
	2. Chronic ischemic stroke	
	3. Severe lower limb ischemia	
Autologous immunotherapy (CIK, DC,TIL, NK,DC-CIK, gamma-delta T cells, and adoptive T cell transplant)	1. Hematological malignancies when standard treatment is ineffective	
	2. Stage 1–3 of solid tumor when standard treatment is ineffective	
	3. Stage 4 of solid tumor	
Autologous adipose tissue derived stem cell transplantation	1. A difficult or chronic wound for six (6) weeks	
	2. Large area of burns or skin damage that total surface is of 20% (inclusive) areas Subcutaneous and soft tissue defects	
	3. Degenerative arthritis and knee cartilage defects	
	4. Combination or adjuvant therapy of superficial minimal invasive technique	
Autologous fibroblast transplantation	1. Skin defects: filling and repair of wrinkles, pits, and scars	
	2. Subcutaneous and soft tissue defects	
	3. Combination or adjuvant therapy for other superficial minimally invasive techniques	
Autologous bone marrow mesenchymal stem cell transplantation	1. Degenerative arthritis and knee cartilage defects	
	2. Chronic ischemic stroke	
	3. Spinal cord injury	
Autologous chondrocyte transplantation	1. Knee articular cartilage	

CAR-T, chimeric antigen receptor-T cell; NK, Natural Killer cell; NKT, Natural Killer-T cells; CIK, cytokine induced killer T cell; DC, dendritic cells; TIL, Tumor infiltrating lymphocyte.

To evaluate the safety and efficacy of the approved CBT products and in consideration of their reported varied efficacy, an annual mandatory review process to monitor adverse events or abnormal side effects has also been included in the regulation. Such review entails the submission of a report covering all treated cases and treatment effects to the MoHW for future amendments to the list of approved CBT products as well as to create public awareness of the risks-benefits for transparency purposes. Thus, the accessed risks-benefits from routine evaluations determines whether an approved CBT product can be turned into a routine medical practice or annulled.

**FIGURE 3:**
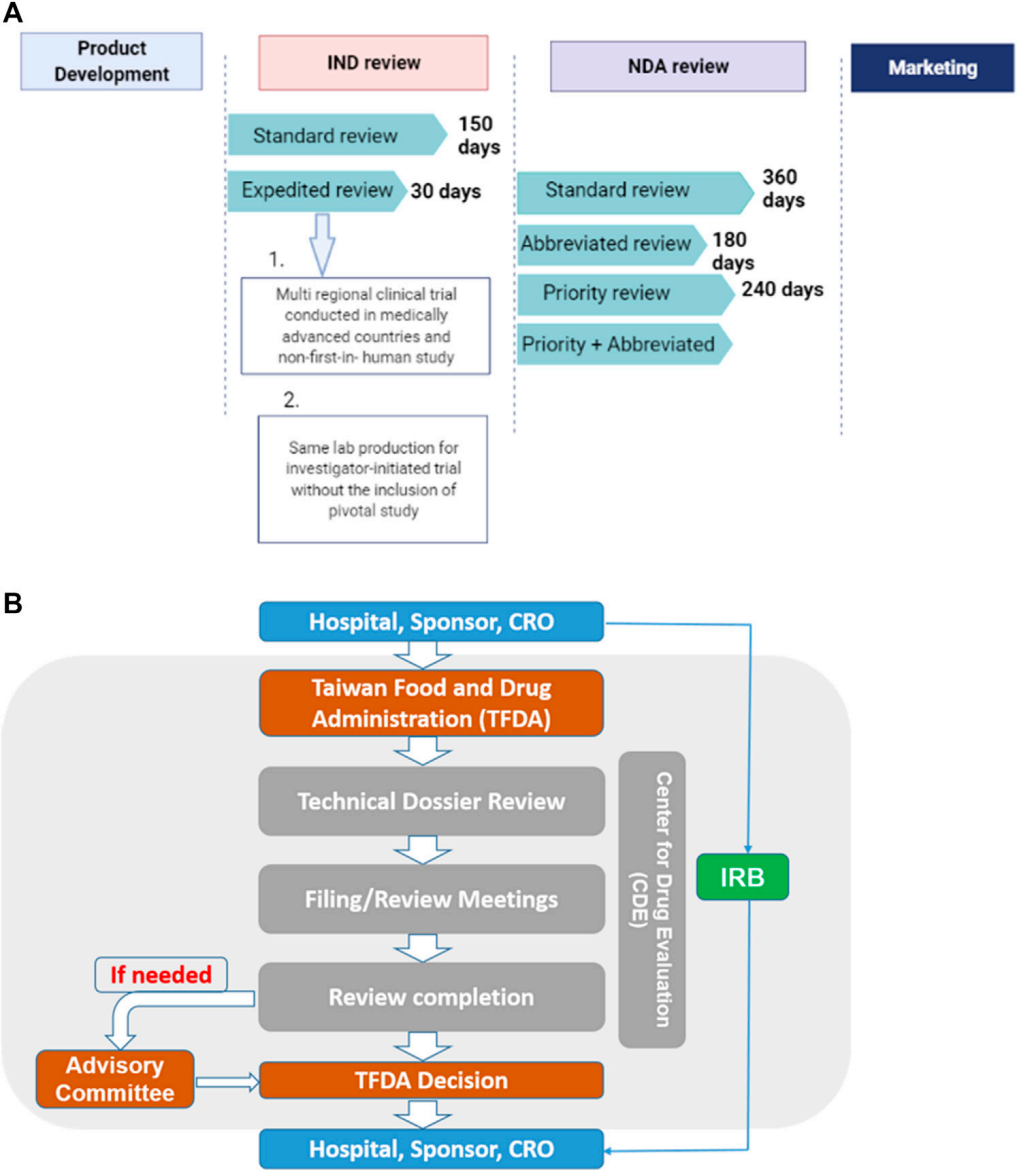
**(A)** The standard IND review takes 150 days while an expedited review takes 30 days. On the other hand, the NDA application, it takes 360, 180, and 240 days for standard, abbreviated and priority reviews respectively.conditions for expedited review **(B)** Sponsors, hospitals and CROs file application through the TFDA. Application is sent to the CDE review section which reviews the technical dossiers for conclusion. Report may be sent to the advisory committee if need be. The overall report from both the CDE and advisory committee will be submitted to the TFDA for final decision making.

The “Regenerative Medicinal Product Act” (still in draft), on the other hand, and was designed to manage regenerative products. Hence, the act governs high risk products including cells, genes, and medical devices (combinations of cells and medical devices). The use of embryonic stem cells in research is governed by the “Code of Ethics for Embryonic Stem Cell Research” and the “Guidelines for Ethical Policy on Human Embryo and Embryonic Stem Cell Research” in Taiwan. However, because induced pluripotent stem cells are derived from somatic cells, it is thus excluded from embryonic stem cell controversies. Therefore, the clinical use or application of induced pluripotent stem cells are thus governed by the regulations on human cell products clinical trial, and Genetic Recombinant Experiment Regulation (FC, 2020).The regulatory authority, the Taiwan Food and Drug Administration (TFDA), oversees the manufacturing of regenerative products under the GMP (Good Manufacturing Practice) standards, and the conduct of clinical trials for regenerative products while the Center for Drug Evaluation provides consultation advice and assists in the review of medicinal products (Figure 3). Thus, the medicinal product act requires an application for both investigational new drugs, and market approval. Furthermore, a distinct pathway which permits conditional approval (for unmet medical needs) of cell-based products for market authorization of 5 years based on safety and preliminary efficacy data. This pathway requires robust clinical data from a phase II clinical trial provided by sponsors, and thus considered for initial approval after which sponsors must conduct confirmatory clinical trials from phase III to validate the clinical benefit otherwise the conditional approval will be withdrawn. There are currently no cell products approved under this act. However, numerous clinical trials are underway.

The government has prioritized biomedicine at the forefront of its biomedical industry innovation scheme to position the country as a hub for biotechnologies (Chen, 2016). This initiative placed significant emphasis on the development of high end therapies including regenerative medicine and cell-based therapies. One such effort is the enactment of the current regulatory framework which includes a complimentary effort that allows for clinical innovation, and was expected to accelerate the development of regenerative medicinal products However, no cell therapy products have been approved for marketing thus far. According to the literature, the slow pace of CBT development may be due to scientific, and legal or regulatory challenges amongst others (Kemp, 2006; Mason, 2007). Hence, to understand this, we explored the opinions of experts collectively involved in medical practices under the purview of the “Special Regulation for Cell Therapy” to understand their perspectives and the challenges to such practices. The resulting perspectives could influence regulatory authorities’ development of strategies that could accelerate the development of cell-based therapies.

## 2 Analysis of the Cell Therapy Projects

**FIGURE 4:**
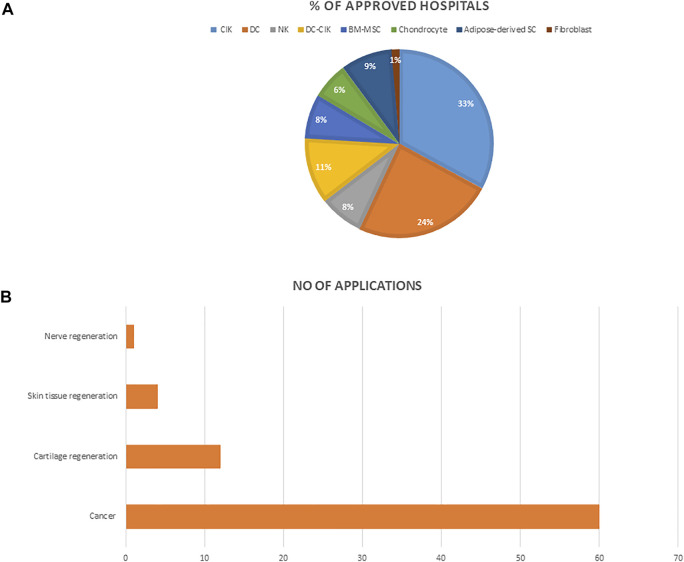
**(A)** The percentage of hospitals approved for each of the cell technologies **(B)** The number of applications approved for each of the indications. Abbreviations: NK, Natural Killer cell; NKT, Natural Killer-T cells; CIK, cytokine induced killer T cell; DC, dendritic cell; BM-MSC, bone marrow mesenchymal stem cells.

We searched and reviewed information on all approved cell therapy technologies under the special regulation for cell therapy including the medical institutions on the Ministry of Health and Welfare Website. We then analyzed the number of approved institutions, the conditions for which these technologies are mostly applied, and the percentage of medical institutions approved to use each of the cell technologies (Figure 4).

## 3 Interviews

Qualitative methodology is well suited for the exploration of complex issues. Thus, we conducted a qualitative study by means of semi-structured interviews to understand respondent’s perspectives on one of the dual-track regulatory framework in Taiwan (Griffith et al., 2017). We followed the Consolidated Criteria for Reporting Qualitative Research (COREQ) guidelines to ensure rigor in our study (Tong et al., 2007). Ethics approval was Granted by the Taipei Medical University Hospital-Joint Institutional Review Board.

Eligible respondents who work in approved academic affiliated-medical institutions and cell therapy industries were recruited by purposive sampling (Patton, 2002). This sampling technique common in qualitative research aims to select participants who can provide rich and in-depth information rather than generate generalizable data. Forty-nine email invitations were sent, and sixteen participants agreed to be interviewed. We aimed to explore expert’s perceptions of the special regulation for cell therapy, the implementation challenges, and respondent’s recommendations. To address our research questions, the interview questions were designed, and organized around these areas. We sent the interview guide prior to our meeting with each of the respondents. All respondents consented to the study through written informed consent, after being provided with the details of the study, and measures for maintaining their confidentiality. Accordingly, 11 in-person and 5 online (telephone) interviews were conducted lasting between 30–60 min each. No incentives were offered to any of the participants. These interviews were conducted individually between February and October 2021.

The interviews were conducted and audio recorded by BAA and subsequently transcribed verbatim before being imported into the data management software ATLAS.ti (Version 8; scientific software Development, Berlin, and Germany) by BAA. BAA and MKS conducted an inductive thematic analysis as described by Braun and Clarke (Braun and Clarke, 2006) to identify themes within respondent’s narratives. According to this approach, the first step entails familiarization with the interview through reading, and re-reading the interview text. During this time, BAA began developing a coding framework which was expanded as needed as the transcript were coded. Then, BAA, and MKS began the analysis, formulating codes to the textual data based on sentences that are relevant to the study. A code book was created and reviewed, as we openly coded each of the interview transcript. We further collapsed the codes into groups with related patterns, and then into larger categories after which BAA, MKS, and CHL met to discuss, agree, and reach a consensus on the categories. Finally, each theme was named and the report was drafted. We ensured the emergent themes are well grounded in the data by critically reviewing each of the themes against the transcripts to uphold credibility. Exemplar quotes were assigned for illustrative purpose.

## 4 Results

An analysis of cell therapy projects indicated that over the past 3 years, autologous cells that involve using a patient’s own cells were approved for use across medical institutions under the special regulation for cell therapy. These cells were categorized based on their level of manipulation. Therefore, the types of cells permitted were; 1) cells that have undergone simple separation and purification steps, and thus implant directly such as Hematopoietic stem cells 2) cells that have been expanded and cultured in GTP approved laboratories. These include immune cells, mesenchymal stem cells, fibroblast, and chondrocyte, etc (excluding genetically modified or combination therapy of cells and devices), which have been allowed for use in tissue regeneration and cancer treatment. (Table 1). As of October 2021, a total of seventy-nine 79) medical institutions have been granted approval to use any of the listed cell technologies most of which address cancer (Figure 3). Furthermore, cytokine induced killer cells (CIK) is the most used, and applied technique amongst all approved medical institutions.

Sixteen respondents participated in the interview portion of this study; including (9 physicians who are specialist in oncology, neurology, pediatrics, orthopedics, and 2 physician’s assistant across 8 medical institutions) (4 experts across four cell processing units and industries) and a review official. Of the sixteen respondents, 13 of them (81.2%) were male and 3 of them were female (18.7%). Most respondents were highly educated with 12 of them (75%) holding PhD degree, and 2 of them (12.5%) holding an MSc degree in addition to their undergraduate degrees. Three major themes and four subthemes emerged from this qualitative analysis: 1) Respondent’s perceptions of the “Special Regulation for Cell Therapy” 2) Emerging issues and controversies on medical use of cell technologies in private clinics, and 3) Challenges impeding the clinical innovation of cell technologies which include the efficacy and cost of autologous therapies, regulatory demands, and clinical outcome evaluation and review.

### 4.1 Theme 1: Perceptions of the “Special Regulation for Cell Therapy”

Most of the respondents commended the TFDA for structuring the current regulation for CBT, as this was considered “new technology” with restricted use in many countries. Respondents understood the regulation to be an indispensable strategy to ensure progressive effort on CBT development in Taiwan, hence the regulation was interpreted as being in a nascent stage. Some of the respondents reported that the initiation of this regulation was influenced by patient advocacy groups. Therefore, the clinical use of CBT was perceived as hope for patients. Hence, the limitation on the approved cells, and intended diseases was impelled by safety concerns to protect patients. Respondents were optimistic about the future of cell therapy with high expectations regarding what seems like an ideal cell therapy treatment as described:

“Cell therapy is the future. It is the future and it is very important. It will be very important in medicine, but it’s just beginning now. I think the TFDA also wants to open this window for cell therapy. However, their concern is the safety, so they will open step by step.” (*Physician*)

Some respondents felt that the preceding cell therapy regulation governing multiple phases of trials limited physician’s hands–on–experiences while others considered it as ‘unfit’ for CBT. Hence, preference was given to the “Special Regulation for Cell Therapy” by some respondents, as it provides an avenue for physicians to develop proficient skills which are essential during clinical trials, as well as those needed for the standardization of cell quality, and the development of smaller biotech companies. On the other hand, others considered the regulation unsuitable for clinical evidence and scientific rigor crucial for CBT development. One respondent expressed concerns over the difficulty that might be faced by the government in balancing the scientific and commercial aspects of CBT, emphasizing that the benefit of CBT to patients should be considered a priority, as clinical evidence is needed to reveal the best treatment for patients. Thus, respondents suggested that clinical trials should be encouraged to provide more concrete scientific evidence for long-term benefits to patients as this quotation clearly notes:

“For promoting practice, for promoting, research, you can do this under this special regulation with this, you cannot really prove [that] your product [is effective]. You [are] just giving people hope I certainly do not reject this special regulation because it’s more like giving people [the] chance to try. So, you can link this to the idea of the right to try argument.” (*Reviewer*)

Some respondents felt Taiwan lags behind other countries in CBT development due to having stricter regulations. Contrarily, others described the regulation as lax owing to the exclusion of rigorous trials. Unprompted, a respondent felt that the government’s decisions on regulatory policies were informed by scientific findings from other countries as described:

“I don’t know why maybe they think it [is]… dangerous to use cells for humans. So, they [are] afraid, they are afraid to allow the company … [to] use them in human. I think the [government used] scientific findings from other countries to support their decisions.” (*Physician*)

Respondents tended to attribute the slow pace of cell therapy development to the strictness of the regulation, as they believed the government is concerned about patient safety.

### 4.2 Theme 2: Emerging Issues and Controversies on the Medical Use of Cell Technologies in Private Clinics

Some respondents perceived the use of CBT in private clinics to be illegal according to the regulations. Contrarily, others felt the regulation permitted private clinics to use these technologies as parts of medical institutions. In an effort to improve oversight on cell therapies, the government recently proposed to allow the practice of cell therapy in private clinics. Yet, whether or not such practice should be allowed in these clinics has remained controversial. Most of the respondent’s perceptions were based on safety and risk management. For clinics to be permitted, most respondents felt the cell techniques should be lower in risk as there are lack of proper facilities or teams to care for patients in case of serious adverse events. However, others agreed with the idea due to a need for clinical evidence to promote the development of CBT. Another area of concern centered on the importance of scientific knowledge on the part of physicians who are involved in private practice, as one respondent described such knowledge as critical to ensure patient safety. Thus, inspection of activities, including the cell processing units and the expertise of physicians to ensure patient safety were suggested.

### 4.3 Theme 3: Challenges Impeding the Clinical Innovation of Cell Technologies

Respondents identified four challenges faced in the clinical development of cell-based therapies in Taiwan.

#### 4.3.1 Subtheme: The Efficacy of Autologous Cell Therapies

The limited types of autologous cells approved for clinical use in medical institutions was considered sufficient by some respondents, while others perceived this to be a strong barrier, and as they hoped cell therapy could be approved for diseases for which conventional treatments are ineffective. From the interviews, it is apparent that the approved autologous cells did not live up to the expectations of both the respondents and patients, and considering that an overwhelming number of respondents expressed their dissatisfaction regarding the treatment outcome having previously treated their patients with autologous cell therapies. One respondent described:

“So, the autologous cells might not be so perfect but this is the only current useable … [cell source] under this regulation.” (*Physician*)

The majority of the respondents who reported dissatisfaction were oncologist. Some of whom perceived autologous cancer immunotherapies as insufficient for late-stage patients, but they predicted a greater chance of success for those in the early stages of cancer:

“I think the challenge [is], for the so called non-advanced cancer, for example, and stage 1 or early cancer. Is it possible to use adjuvant cell therapy? Is it rational? The solid cancer has no guideline [for] cell therapy use [in] HCC [hepatocellular carcinoma], breast cancer and pancreatic cancer, and there is no such suggestion. So, I think this is the challenge. If patients [those in the early stag] say, I have money, I want to do cell therapy, can you ... [Offer me]? I say no”. (*Physician*)

Both physicians and industry experts recognized that the efficacy could be influenced by factors such as cancer types and status, the patients condition (e.g., age, compromised immune system), and chemotherapy interference, which could also influence the quality and freshness of CBT as described by a respondent.

“Because the cancer patients have been treated with chemotherapy … you cannot collect enough of [their] blood … sometimes the lymphocyte quality … or NK [Natural killer cells] ratio is very low... In that case, it is very difficult for us to get [a] very good quality product.” (*Industry personnel*)

Thus, current methods used to address the cell processing issue involves close team work with physicians for proper timing of chemotherapeutic treatments in the case of immunotherapies, and as some of these cells do not have the ability to tolerate the condition of freezing and thawing. As such, they do need to be freshly cultured to maintain their quality. Cells derived from adipose tissue, however, were stated to have standardized procedures of extraction but quality control still remains a challenge in developing all of these products. Furthermore, the uncertain efficacy led some of the experts to seek alternatives as well as influence government decisions on amending approved cells. With this, some respondents were optimistic about chimeric antigen Receptor-T cells and allogeneic cells, considering their efficacy outcome and approval for certain indications in other parts of the world. However, others cautioned about the risk of these alternatives. Finally, many of the respondents suggested that the authorities need to be open-minded by approving more cell types, and disease indications when conventional treatment is ineffective, while also being cautious.

#### 4.3.2 Subtheme: Clinical Outcome Evaluation and Review

Respondents revealed that registries were created to monitor the practice of cell therapy which could benefit regulatory decisions on approved cell therapies. They indicated the necessity of monitoring adverse events and efficacy of regenerative products, as cell therapy is still immature. One of the respondents described:

“I think it’s necessary [annual review mechanism] because actually [whether] it is cell therapy or product … it is not mature right now. So, even a pharmaceutical drug has what we call phase IV [clinical trial] after you sell the product, and you have to [review what was approved].” (*Physician*)

Preference was shown for electronic collection of data with emphasis on its usefulness in building data banks, which could, in turn, promote Taiwan’s global competitiveness in terms of data storage. The electronic case form was designed for specific diseases. Hence, some respondents expressed concern over the design due to its tendency to introduce statistical bias, and particularly for stage 4 cancer patients who had received different standards of care before CBT. Simplification and specification of electronic forms were suggested to enhance statistical analysis for better efficacy evaluation. In addition, the establishment of a suitable electronic reporting systems by stem cell companies was suggested, and as the form was described as being in its infancy. A respondent stated:

“It is better to ask patients or doctors to report their practice … It’s better to have a special form like the electronic forms so that we can report everything and once you get this data you can analyze every doctor’s results [and] patients’ benefits. The report form is [in the] very early stage.” *(Physician)*

The difficulty of characterizing the effectiveness and analyzing outcomes in combination treatments of chemotherapy and autologous cell transplant were also a concerns raised by respondents. Hence, they were skeptical about the success of the review mechanisms as they believed it might not give a clearer endpoint akin to clinical trials. There were, however, contrasting opinions as to whether this challenge exists in clinical trials particularly in oncology. For some respondents, the idea of the annual review mechanism gives the impression of a clinical trial due to the detailed record-keeping and supervision involved. However, this perception was contrasted by other respondents who indicated that stringency is necessary. There were contrasting opinions as to how long review follow-ups should be done to relieve the government from frequent reviews despite the consensus on the need to review the clinical use of CBT. The lack of resources and support in data collection was considered a challenge, rendering the process onerous and potentially even detrimental to the field as this respondent described:

“Because it’s a new field for [the] hospital, even though we have doctors, nurses and we have some cancer registry staff but everybody is not familiar with cell therapy and … what you have to do [is not understood by everyone]. So, everybody is new in this field … and there seems [to be] a lot of things to do and there seems to be a lot to report[s]. So, not so many people want to touch this field.” *(Physician)*

However, another respondent indicated that study nurses responsible for data collection also act as middlemen between doctors, companies, and patients. The respondent, however, did not comment on how this arrangement may affect the nurses and their other responsibilities.

#### 4.3.3 Subtheme: Cost of Autologous Therapies

Many respondents perceived the cost of autologous cell therapy as burdensome to patients. Some respondents felt the source of starting material of CBT determines the cost of cell therapy. However, others considered the high cost of autologous therapies to reflect the cost of all new drugs. Many respondents believed that high the cost of cell therapy was a result of the manufacturing standards and the patient-specific nature of autologous treatments. A respondent commented that high cost could be justified only if autologous therapies were more effective but currently, these therapies are still in the experimental phase. Currently, however, unaffordable prices may deter patients from seeking cell therapies as patients are not accustomed to paying out of pocket because Taiwan has a national health-care service that covers the majority of patients expenses. Furthermore, some respondents felt the national health care services was a poor adopter of innovation. A respondent described:

“In Taiwan, I think it’s very difficult to make progress under this insurance because there’s no support for innovation because innovation needs money” (*Physician*)*.*

Some respondents believed the consequence of high manufacturing standards could lead to the proliferation of unregulated practices amongst companies abetted by patients who may not be able to afford these therapies. Others seemed hopeful for government strategies, industry competition, mass production of allogeneic cells and insurance coverage to address the high costs of these therapies.

#### 4.3.4 Subtheme: Regulatory Demands

Medical institutions have no experience and experts to handle regulatory affairs. Moreover, a respondent mentioned that regulatory personnel in biotech companies have little knowledge about regulatory compliance. Therefore, respondents indicated that there is a need to incorporate regulatory studies for science students across institutions.

“The regulatory education is not in the system basically. I don’t know if it is there outside of Taiwan but in Taiwan, we basically learn zero knowledge [about] regulatory compliance or something like that … So these people [life science and biology students] enter industry and solely become the manager or leader of the company but they don’t really have the tools or the sense for the regulation.” (*Industry personnel*)

Regulatory demands were perceived as difficult which often lead to disputes between cell developers and the regulators. It is believed that inadequate scientific knowledge about innovative therapies due to the rapid pace of their developments, in addition to the small market size, and the lesser number of applications reviewed as compared to their international counterparts (i.e., the USFDA) could be a reason for the longer approval times for the clinical testing of products. On the other hand, under the context of the special regulation, some respondents felt that the frequent testing and validation for autologous cell processing, and the use of GMP clinical-grade reagents are expensive. They acknowledged the enormous reviewing responsibility of the review committee on applications from various medical institutions but raised a concern on the criteria used in evaluating each of the applications. However, the challenges faced by the reviewers centers on the difficulty to evaluate qualified protocols and the measure of preparedness of physicians and their cooperated companies. Thus, reviewers are skeptical about what to approve as described:

“We are also not sure what will happen if it is really applied to the patients ... Could there be any harm, serious side effect or not effective at all and patients complain … So, things like that could happen, we can imagine … or the patient got this treatment then has some infection, some serious side effect, things like that.” *(Reviewer)*

Regulatory ambiguities and the probing nature of regulatory officials was considered a negative influence on their relationship as they audit their cell processing units and procedures during site inspections. Some respondents believed the major concern is safety which compelled regulators and reviewers to protect patients from harm while focusing on benefits. However, one respondent from the industry revealed the continuous effort of regulators to raise the manufacturing standards, such as from GTP to GMP but cautioned it could stall commercial development of CBT due to the high cost of maintenance. Therefore, respondents indicated that the challenges faced by regulators and reviewers include; balancing patient safety and the commercial development of CBT which impacts the time it takes to evaluate and approve applications. A respondent described their challenge with the review committee:

“[A] committee needs to check your project, it takes a long time, and sometimes the committee [challenge applications the same way when you] submit your paper [for publication]. Even, if you have nothing … [that should be challenged] they... [feel the] need to challenge something.” (*physician’s assistant*)

Training and recruitment of regulatory staff who understands critical points about CBT was suggested by some respondents. Others emphasized the need for regulatory clarity, educational resources on regulatory standards, early communication, and shorter approval times.

### 4.4 Regulatory Comparison: Learning From Other Jurisdictions

In the United States, the United States Food and Drug Administration (US FDA) established a three-tiered regulatory framework, which indicates an increasing level of oversight as the risk of CBT increases, falling either within the “351 or 361” category based on the Public Health Service Act, and 21 Code of Federal Regulation (Table 2) (U.s.f.a.d. Administration, 2007). The regulation exempted and designated 361 products as minimally manipulated cells, which only require listing and registration with the FDA. Similar to the United States, the EU has also adopted a risk based approach by exempting minimally manipulated cells regarded as “cells intended for human application” under the Directives 2004/23/EC (Commission Directive (EU), 2015). However, unlike the 361 products in the United States, they do not require listing and registration. The EU also have an additional exemption known as hospital exemption (HE) which permits the use of advanced medicinal therapy products across medical institutions.

**TABLE 2:** Regulatory model for clinical innovation across different jurisdictions.

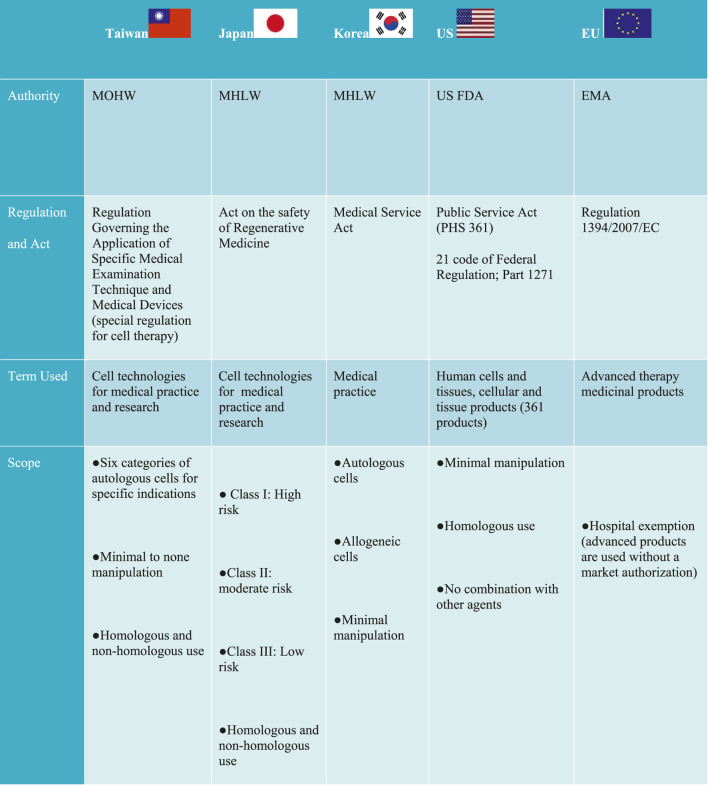

MoHW, Ministry of Health and Welfare; TFDA, Taiwan Food and Drug Administration; MLHW, Ministry of Health, Labor Welfare; MFDS, Ministry of Food and Drug Safety, USFDA, United States Food and Drug Administration; CAT, Committee for Advanced Therapies; EMA, European Medicines Agency.

Contrary to the United States and EU regulatory frameworks, Japan, Taiwan, and Korea use a dual track regulatory framework to regulate CBT for medical practice and product Japan permits the use of processed cells (more than minimally manipulated cells) and does not exclude non-homologous use in medical practice under the Act on the Safety of Regenerative Medicine (ASRM) (Table 2). Unlike Japan, Taiwan approved only 6 categories of minimal to none manipulated autologous cells for specific indications under the special regulation for cell therapy (CHEN, 2019). However, similar to Japan’s ASRM, non-homologous use was not excluded. The use of minimal manipulated cells from both autologous and allogeneic sources was approved for medical practice in Korea. However, the health authorities deems it fit to consider minimal manipulated cells as products requiring a pre-market authorization; if the manufacture of such cells take place outside of medical institutions (Han and Shin, 2015). In China, cell therapy is regulated in the broad category of stem cell therapy regulated under the Administrative Measures of Clinical Research for Stem Cell (AMSC 2015) or somatic cell therapy regulated under the Management of Clinical Research and Transformation Applications for Somatic Cell Therapy MSCT 2019 (Wu et al., 2020). Thus, both stem cell therapy and somatic therapy independently have a dual track framework for clinical research and clinical application in medical institutions as well as market authorization for biological products, which is jointly regulated under the Guidance for Research and Evaluation of cellular therapy products (Wu et al., 2020).

In general, substantially manipulated cells are considered products and are regulated differently in all of these jurisdictions, but all commonly require clinical testing. Furthermore, market authorization also varies in all of these jurisdictions. For example, a time-limited condition approval for market authorization is required in Japan (7 years) (Konomi et al., 2015), Taiwan and Korea (5 years) (Lim, 2015; CHEN, 2019).These countries also have similar pathways initiated to expedite the approval process of CBT but differ in their names and prerequisite. Regardless, designing a suitable regulation to accommodate CBT as well as the harmonization of such effort has been a global challenge (Aly, 2020). Considering the position of the United Kingdom and Japan as front runners in the development of regenerative medicine, Taiwan can improve their process in the areas of infrastructure and human capital development described as follows:

#### 4.4.1 Establishment of physical infrastructure

Currently, there are no large-scale manufacturing plant to cater for RM. Taiwan could learn from the United Kingdom by providing more physical infrastructure which would help enhance RM. Although a plant is being built in Hsinchu (Intralink 2020), more would be essential with numerus manufacturing clean rooms to increase manufacturing capacity. This would make it easier for companies to hire these plants rather than having to build their own GMP facility. In addition, regional hubs or centers similar to the United Kingdom Advanced Therapy Treatment Centers (Umemura and Morrison, 2021) could be established to promote clinical delivery and foster a mutual collaboration between clinicians, biomedical scientists and small biotech companies.

#### 4.4.2 Human capital development

Since skill development is crucial for the development of RM, it is essential to focus on improving human capital. Similar to the United Kingdom and Japan, Taiwan can also incorporate courses and certification that includes cell culture techniques, bioethics, and safety for specialist or technologist in RM.

## 5 Discussion

This study explored the views and concerns of experts on the special regulation of CBT and the challenges hindering medical practices in Taiwan. Despite recent reform of the regulatory framework, respondents still perceive the development of these therapies as challenging. Having implemented the special regulation, some felt its conservative feature which permits only six (6) types of autologous cells and the limited indications have impacted the progress of such development. They also have varied opinions regarding the level of oversight needed by the health authorities which they assume contributes to the pace of these developments. Some perceived the regulation as strict having considered the development status in other countries in addition to the quality standards imposed by the authorities. Others felt it is lenient as scientific evidence is vital to these developments. But for most of the respondents, the regulation was considered experimental, and as such they emphasized the need for improvements and hoped to accumulate more experience as they continue to develop cell-based products.

The previous regulatory model which considered cell therapy as product without any other expedited developmental pipeline as well as the lack of funds to complete a product life cycle was regarded as challenging by many cell therapy developers (Plagnol et al., 2009; Dodson and Levine, 2015; Chen et al., 2017). Additionally, many patients who do not fit into trials were known to seek unregulated treatments from other countries (medical tourism) which poses serious risk to patients and lack of follow–up evaluations (Matthews and Iltis, 2017). To protect the public from potential harm, the special regulation addresses: 1) the issue of stem cell tourism by promoting patient access to cell treatment for diseases for which conventional treatments are ineffective; 2) safety concerns by implementing annual adverse event reporting mechanisms to promote accountability for potential risk from cell-based treatments; and 3) boosts the experiences and proficiency of physicians on the technical know-how of transplantation procedures as well as help regulatory officials in gathering more experiences on proper risk assessment. Additionally, it lowers the cost of development for smaller companies to promote their development.

The concerns raised in other jurisdictions were mostly about ambiguities and definitions around minimal manipulation and non-homologous use-particularly where some of the regulatory agencies drew distinctions around the definitions of these terms (PEW, 2019). For example, the United States FDA’s distinction between structural, and non-structural tissue led to the consideration of adipose-derived stem cells as drug products rather than in the 361 category as expected by cell therapy developers (PEW, 2019). Such differing definitions and the scope of minimal manipulation across jurisdictions amid the growing market of unproven stem cells in private clinics has been reported (Turner and Knoepfler, 2016). In order to improve the oversight on CBT in Taiwan, a key question and an emerging issue was whether private clinics should be approved to offer CBT within the context of the special regulation. This question is an ongoing debate; as recent reforms consider approving private clinics to offer CBT. Respondents opinions on risk management were based on the lack of proper teams and facilities to care for and follow up patients. Such a zero-risk stance is understandable in light of risky private practices that have caused harm to patients in the past (Lomax et al., 2020). In addition, the lack of scientific knowledge pertaining to CBT could influence their interactions with patients (Smith et al., 2021). These concerns corroborate a previous study which revealed that patients depend on the judgement of physicians and see them as reliable sources of information regarding CBT (Aiyegbusi et al., 2020). On the other hand, the lack of clinical evidence base is the reason why some respondents were in favor of cell therapy practice in clinics considering the need for supplementary clinical evidence on the approved autologous therapies. In line with this, the Taiwanese regulatory authorities require strict inspection of quality standards for cell processing and facilities, follow-up record system and co-operation with medical centers specifically for private clinics interested in using cell based technologies as cancer treatment.

The concerns regarding the special regulation expressed by respondents include: 1) its inaptness for clinical evidence; 2) the limited approved cell treatments and indications; and 3) the time and criteria used by review committees to assess and approve applications. Similarly, the European Hospital exemption (HE) of all the regulatory strategies has been an area of concern among stakeholders due to its different interpretations, and implementations across European member states (Hourd, 2014). Thus, there are growing expectations for the regulation to be improved considering that the clinical data from HE could help Advanced Therapy Medical Products development. To address concerns about clinical evidence in Taiwan, health authorities recently announced their intent to use real-world evidence to bridge evidence gaps between cell therapy techniques and products. To this end, clinical data obtained from medical practices could be used as supporting evidence to develop medicinal products as incorporating real world evidence could reveal the efficacy of CBT treatment. A similar idea was recently proposed for Japan’s ASRM to transform the clinical practice of cell therapies into an evidence-based form (Takashima et al., 2021). Such a practice is also being considered in the 21^st^ Century Cure Act of the United States FDA for new indications and post-market surveillance of regenerative medicine advanced therapy designated products. How this will be done has not been clearly stated by the regulatory authorities in United States or Taiwan. Thus far, Taiwan had successfully used real world evidence to inform regulatory decisions on the approval of pharmaceuticals, i.e., sapropterin tablet, oral ketoconazole, and alteplase (Chen et al., 2020). Therefore, such experience might prove useful in addition to experience sharing and learning from other jurisdictions who have considered or may be considering the use of real world evidence (Earl, 2019; Takashima et al., 2021). Moving forward, there is need for more clarification from the Taiwanese health authorities on what is expected of cell therapy developers regarding the use of real-world data.

The challenges hindering medical practice highlighted by respondents were, first, autologous cells appear more pleasing owing to their minimal risk of rejection as compared with allogeneic cells. Yet, it is evident that physicians and industry experts were dissatisfied with their efficacy outcomes. This is in line with the literature considering efficacy is patient specific and largely influenced by a number of factors (McAllister et al., 2012; Hurley et al., 2018; Krause et al., 2019; Du et al., 2021). Furthermore, this dissatisfaction may be predictable considering that those approved cells were intended for life-threatening diseases such as end stage cancer. With this, we observed optimism on the part of physicians, as they seek alternatives to help their patients, and likewise companies have shown willingness to develop allogeneic cell therapies (Plagnol et al., 2009).

Secondly, clinical outcome evaluation was regarded as problematic as the design of the electronic form was revealed as inapt for efficacy evaluation in addition to concerns about how to measure efficacy outcomes. Also, the reported fewer number of patients enrolled based on MoHW database could result in a wide evidence gap and the need for more clinical evidence. This is in line with a previous study that revealed limited evidence base and developmental pipeline in the regenerative field, especially in patients with rare diseases (Abou-El-Enein and Hey, 2019). The idea of clinical data collation in preparation for review was perceived akin to clinical trials and regarded as complicated compared with routine medical practice. This is in line with the study by Takashima et al. (Takashima et al., 2021) which indicated the challenges of data collection for clinicians (Takashima et al., 2021). Thus, the unwillingness of healthcare professionals could lead to a shortage of clinicians, and hence slow the pace of CBT innovation in Taiwan. Our findings suggest the need to fund and support clinicians who are invested in fulfilling the role of data collectors and other related duties.

Thirdly, the large out-of-pocket costs were indicated as burdensome to patients as shown by the fewer number of enrolled patients. The implication is that only those who can afford the therapies will have access which could, in turn, lead to access inequality (Aiyegbusi et al., 2020). The contributing factors to the high-out of pocket costs include the patient-specific nature of autologous cell therapies and manufacturing standards which were considered problematic for hospitals and small companies (Gee, 2002). Although, mass production of allogeneic cells was considered cost effective, however, it is possible that the cost of manufacturing, and regulatory oversight by the TFDA refute such theoretical statement (McAllister et al., 2012). Accordingly, the government is trying to control and negotiate the prices of these therapies with both the companies and medical centers but how this negotiation will be done in the face of the GMP manufacturing standards is unknown.

Finally, we observed tension between reviewers and these experts as they noted their inability to understand their expectations on regulatory demands and standards. Moreover, United States FDA officials were considered more experienced. This is not surprising considering the larger market size, hence, and broader familiarity with innovative therapies on the part of their regulators and reviewers. It turns out that the regulatory standards were perceived to be very demanding making it difficult for medical institutions and small companies to navigate the regulatory environment. Furthermore, lack of familiarity and understanding of regulatory compliance have complicated the situation as medical institutions do not have offices for regulatory affairs in addition to the limited knowledge about compliance in small companies. Currently, medical institutions in other countries are beginning to open up regulatory offices to cater to cell therapy developments. On the other hand, it is largely difficult for regulators across jurisdictions to keep up with these innovative therapies, given the field of cell therapy is evolving at a rapid pace. In light of this, the reviewer's probing nature to evaluate the risks and benefits is understandable, since there are no tools to measure the risk-benefit ratio (McAllister et al., 2012). Therefore, the challenge of how to properly review CBT in terms of safety, quality, and efficacy without stifling the development of CBT (Lysaght et al., 2018) is not an easy task to manage but not insurmountable either.

Our study provides key insight into experts perceptions of the “Special Regulation for Cell Therapy” in Taiwan and challenges to the clinical development of CBT. The strength of the study lies in the differing backgrounds of respondents allowing for some degree of reflexivity and nuanced insight. All respondents are very knowledgeable in their area of expertise as is evident in their level of education and experience in cell therapy development. Respondents were interviewed based on their own point of view, independent of their workplace. Our study does not represent the perspectives of all stakeholders. Therefore, we suggest the need for future research into the perspectives of patients and regulators.

## 6 Conclusion

This study aimed to understand Taiwan’s regulatory framework for cell therapy development. Thus, we focused on experts perspectives and specifically on the implementation of one of the regulatory frameworks known as the “Special Regulation for Cell Therapy”. It is well known that the potential breakthrough of cell-based therapies is fraught with numerous challenges. In spite of this, Taiwanese health authorities has been making tremendous efforts to approve its first regenerative medicine product(s). While the establishment of the new framework was designed to shorten lengthy developmental timelines for the commercialization of regenerative medicinal products, it also addresses safety concerns, patient access and stem cell tourism. Thus, these experts offer insight into some of the remaining challenges especially in relation to evidentiary standards which they believe slows down the pace of these developments. Therefore, to exploit the full potential of cell therapies, there is need for further improvement by the health authorities. It would be helpful for the authorities to adopt policy interventions such as provide funding, educational resources, training on quality control standards, and cost-saving strategies to address some of these unsolved issues as well as communicate with cell therapy developers in a timely manner.

## 7 Summary

### 7.1 Overview of Taiwan’s regulatory framework for regenerative medicine

• The Taiwanese government enacted a dual track framework for regenerative medicine in an effort to position the country as an Asian hub for biotechnologies. The first segment of the framework also known as the special regulation for cell therapy governs the clinical use of experimental cell therapy technologies by physicians while the second segment governs the commercialization and marketing approval of regenerative medicinal products. Till now, no cell therapy products have been granted approval for marketing.
• We interviewed experts from both medical institutions and industries to understand their perspectives on the special regulation for cell therapy and challenges to such practices.

### 7.2 Perceptions on the special regulation for cell therapy

• Respondents felt the regulation provides an avenue for the development of proficient skills in clinical delivery, manufacturing standards and industrialization
• Lack of clinical evidence and scientific rigor was recognized as a challenge.
• Regenerative medicine in Taiwan was perceived as lagging behind other countries.

### 7.3 Challenges hindering clinical innovation

• These challenges included the efficacy and cost of autologous therapies, regulatory demands, adverse event reporting and clinical outcome evaluation.

#### 7.3.1 Efficacy

- The efficacy of the approved six cell technologies used by physicians were a concern to many.
- Factors influencing treatment outcome are cancer status, age, chemotherapeutic influence and the quality of cells.

#### 7.3.2 Clinical outcome evaluation and review

- The current design of case forms for data collection may introduce bias especially for patients in advanced stages of cancer who had received previous treatment.
- The man-power in collating clinical data was considered problematic.

#### 7.3.3 Cost

- The cost of these technologies were considered burdensome to patients as Taiwanese are not accustomed to paying out of pocket due to the National Health Insurance coverage.

### 7.4 Policy

Policy interventions suggested by respondents are funding, educational resources, training on quality control standards, and cost-saving strategies.

## Data Availability

The raw data supporting the conclusion of this article will be made available by the authors, without undue reservation.
